# The comparison of serum interleukin-6 of mothers in vaginal and elective cesarean delivery

**Published:** 2014

**Authors:** Mohsen Haghshenas Mojaveri, Iraj Mohammadzadeh, Zinat Al-Sadat Bouzari, Zahra Akbarian Rad, Ghamar Haddad, Reza Alizadeh- Navaei

**Affiliations:** 1 Non-Communicable Pediatric Diseases Research Center,Babol University of Medical Sciences, Babol, Iran.; 2 Infertility and Reproductive Health Research Center, Babol University of Medical Sciences, Babol, Iran.; 3 Babol University of Medical Sciences. Babol, Iran.; 4 Molecular and Cell Biology Research Center, Mazandaran University of Medical Sciences, Sari, Iran.

**Keywords:** Vaginal delivery, elective cesarean section, interleukin-6

## Abstract

***Background: ***Interleukin-6 (IL-6) has a major role in hematopoiesis, immune and acute phase response and its serum level seems to be associated with the mode of delivery. The purpose of this study was to compare the level of IL-6 in mothers who delivered via cesarean section and vaginally.

***Methods:*** This cross-sectional study was done on 40 mothers with vaginal delivery and 40 mothers with elective cesarean delivery in 2012 in Ayatollah Rouhani Teaching Hospital in Babol, northern Iran. Five ml blood samples were taken from each mother. Blood samples were examined by enzyme-immune assay for the determination of IL-6 in both mothers and neonates. Other information, such as the mother's age, newborn sex, gravity, parity, the number of live births of each mother, and the status of infants in terms of being term or pre-term, was recorded.

***Results:*** The mean age of cesarean section and vaginal delivery patients was (29±5.01) yrs and (27.3± 4.93) yrs. The maternal IL-6 concentration in vaginal birth (170.13 ±15.9) was significantly (p<0.05(higher than cesarean section (33±29.94)

***Conclusion: ***The result shows that serum IL-6 levels in patients with vaginal delivery was higher than those with elective cesarean section delivery. So, vaginal delivery is preferred for all patients except those in whom vaginal delivery is contraindicated.

Cytokines such as IL1B, IL-6 and TNFα play an important role during term and preterm labor. These elements stimulate prostaglandin biosynthesis; therefore, it can cause the stimulation of the uterine myometrium contractions and cervical opening (1). IL-6 is produced through stimulated monocytes in the blood stream (2). The monocytes obtained from neonatal after stimulation with lipopolysaccharide (LPS) are able to produce sufficient amounts of interleukin (IL-6) (3). Higher levels of IL-6 in the mothers cause the appearance of more IL-6 in breast milk. The abundance of interleukin 6 in breast milk can be effective in reducing the incidence rate of neonatal infections and allergies and also, the higher levels of serum cytokines improve the mother’s immune status in the first three months after delivery (4).

The ability of mother and infant for the production of these pro inflammatory cytokines, enables the newborn to deal with infections after birth, while the inability in the production and amplification of their level causes an increase in morbidity and mortality. Also, it can *cause* an *increased* risk of early neonatal infection, necrotizing enterocolitis, bronchopulmonary dysplasia (BPD) and hypoxic-ischemic brain injury (5). 

In a study conducted by Malamitsi-Puchner et al. entitled “A survey of the type of delivery on circulating cytokine concentrations in the prenatal period “, 52 vaginal births and 26 cesarean sections were studied .The result of this study showed the amounts of IL-6, IL-1ß, interferon gamma and TNF-α in mothers and infants of vaginal birth group was significantly higher than the mothers and infants of cesarean delivery group (6). 

The study result of Duncombe G et al. showed the levels of IL-6 and TNF-a in vaginal delivery which was higher than cesarean delivery (7). Global studies are very limited in this field. Therefore, the aim of this study was to compare the serum IL-6 level in two groups of mothers, the vaginal birth and elective cesarean delivery. And to compare them, if the serum IL-6 level was significantly higher in vaginal delivery than cesarean section, vaginal delivery should be performed for all patients except for those whom vaginal delivery is contraindicated. 

## Methods

This analytical study was performed as cross-sectional study on women with vaginal birth and cesarean section delivery in Ayatollah Rouhani Hospital, Babol University of Medical Sciences during 2012. Eighty pregnant women were studied, 40 pregnant women had vaginal delivery and the rest elective cesarean delivery. At alpha: 0.05 and Beta: 0.8, a sample size of 40 cases per group was calculated. In this study, vaginal delivery is a delivery in which mothers enter the labor phase and babies are born with continued uterine contractions without the induction of labor, while cesarean delivery is done as elective and mothers do not enter the labor phase and also induction was not present. 

This study was carried out on samples with the following conditions: Mothers with the parity of 1 and 2, singleton pregnant, term delivery, uncomplicated pregnancy, no underlying diseases such as hypertension, diabetes, cardiovascular disease, a history of drug and alcohol use, and smoking. Exclusion criteria included history of chorioamnionitis, fever, PROM, use of corticosteroid by mother’s maternal illnesses and the use of medications that affect the immune system

At the time of recourse, the maternal blood samples of women with vaginal delivery were obtained just before delivery (in the first stage of labor) when the cervical dilatation was between 6-4 cm. The neonatal samples were obtained just after delivery. The blood samples of mothers who had elective cesarean delivery were obtained just before anesthesia. The collected samples after adding the anticoagulant substance were maintained in the maternity and operating room in a 4-8 °C temperature. The samples were transferred to the lab of Ayatollah Rouhani Hospital for centrifugation and the daily separation of blood serum, and then were maintained at -20°C temperature. The collected blood samples were evaluated by enzyme-immunoassays method in the cellular and molecular unit of the university. The used kits were the products of Austria manufactured by Bendermed System Company. 

Other information, such as mother's age, newborn sex, gravity, parity, the number of live births of each mother, and the status of infants in terms of being term or pre-term, was recorded. 

The collected data were analyzed by SPSS statistical software, data distribution was determined by Kolmogorov–Smirnov test. IL-6 and gestational age in two labor groups were not in normal distribution and Mann-Whitney test was used to compare the amounts of IL-6 and gestational age in two labor groups. A p-value less than 0.05 were considered as statistically significant level.

## Results

In this study, 80 pregnant women were studied, 40 had vaginal delivery and the other 40 elective cesarean delivery. The twenty-one cases of vaginal delivery group (51.5%) gave birth for the first time and 19 cases (47.5%) the second time, while 8 cases of the elective cesarean delivery group gave birth for the first time and 32 other cases (80%) the second time. Eighteen cases of vaginal delivery (45%) were males and 22 other cases were females (55%), While 19 cases of cesarean delivery (47.5%) were males and 21 cases (%52.5) were female. 

The average age of mothers in vaginal delivery group was 27.3±4.93 years and in cesarean delivery group were 29±5.01 years. The average of gestational age at vaginal delivery was 38.4±1.08 weeks and in cesarean delivery was 38.6±0.65 weeks (P=0.38). The results showed that both vaginal delivery and cesarean delivery groups had no significant difference in the average of gestational age.

In both the boy and girl gender, the IL-6 values in mothers with vaginal delivery were significantly higher than the values in mothers with cesarean delivery (P<0.05) (table 1, 2). The mean of neonatal IL-6 concentration in vaginal delivery (10.9) was higher than cesarean section (6.6), but this difference was not significant (p=0.86).

**Table 1:** The mean and standard error of IL-6 values of mothers ​​according to the type of delivery

**p-value**	**MEDIAN**	**Mean±SE (Pg/ml)**	**Group**
	115.05	170.13±15.9	Vaginal delivery
0.008	24.41	33±29.94	Cesarean section

**Table 2:** The mean and standard error of IL-6 values in mothers with vaginal delivery and cesarean section according to the sex of babies

**p-value**	**MEDIAN**	**Mean±SD**	**Group**	**Sex**
0.002	119.47	144.01±133.02	Vaginal delivery	boy
	30.27	37.96±30.19	Cesarean delivery	
0.001	133.17	192.33±177.62	Vaginal delivery	girl
	25.91	33.69±31.97	Cesarean delivery	

## Discussion

The results of our study showed that serum IL-6 concentration in mothers who had vaginal delivery was higher than in mothers with cesarean delivery, and there was significant relationship between the type of delivery and maternal serum interleukin-6 concentration. In the study of Malamiti-puchner et al. entitled "A survey of the type of delivery on circulating cytokines levels during the perinatal period", 52 vaginal births and 26 cesarean deliveries were studied in 2005. The result of this study showed that the levels of serum IL-6 in mothers and infants of vaginal delivery group were significantly higher than its serum levels in cesarean delivery method (6). In a study conducted by Berner et al. in Italy in 1998, the levels of serum gamma interferon , TNF-a , IL-6 , GM-CSF, and the production of blood superoxid (${\overset{\text{¨}}{o}}_{2}$) were examined .The result of this study showed that the level of IL-6 and interferon gamma in mothers who gave birth vaginally was higher than the mothers that gave birth via cesarean section procedure, and the plasma level of blood IL-6 and the production of blood superoxid (${\overset{\text{¨}}{o}}_{2}$) in infants blood delivered vaginally were higher than the babies born through cesarean delivery procedure. Also, there was a significant relationship between the levels of plasma interleukin-6 in cord blood of these two groups of newborn infants. These researchers concluded which delivery method plays an important role in the regulation of host defense system in newborns (8). 

A new theory is presented in this context that physical stress during vaginal delivery can cause the activation of the network of the physiological processes in the mother’s body leading to the secretion and increase of the level of serum inflammatory cytokines. These cytokines can cross the placenta and transfer it to the baby which can be effective in preventing early and late infections of the neonates. While this network is terminated prior to activation because of the cesarean delivery done by a surgical procedure (9). Winkler et al. in a survey on 71 pre-term patients who were born by non-elective cesarean delivery showed the average concentration of IL-1b, IL-6, IL-8 in mothers with cervical dilatation of 2- 4 cm was higher compared with those with dilatation under 2 cm, while the duration of labor and parity did not correlate with the concentration of secreted cytokines (9). 

In a study conducted by Zanardo et al. in Italy in 2006, 25 babies born via vaginal delivery and 25 babies born by caesarean delivery were evaluated that showed IL-6 concentration in umbilical cord blood of newborns delivered by cesarean delivery were significantly lower than the babies born by vaginal delivery (10), While the results of our study are similar to the result of this study, there are also studies that show different results and represent an increase of serum interleukin-6 in cesarean delivery method in a study conducted by Hebisch et al. on 38 healthy pregnant women. The results showed that serum IL-6 levels increased at the onset of labor and declined on the third day after delivery, however, the level of serum IL-6 after cesarean delivery was significantly higher than its level in vaginal delivery (11). Some studies also showed that there were no significant differences between the two types of delivery in the concentrations of serum IL-6. So, in a study conducted by Fukuda et al. on 25 cases of vaginal delivery and 13 cases of cesarean delivery, the serum IL-6 concentration of umbilical cord of neonatal was not significantly different between the two groups (12). 

Also, the result of a study conducted by De Jongh et al. showed there was no significant relationship between the type of delivery (i.e elective cesarean delivery and vaginal delivery) and interleukin levels (13). Also, in the study of Takahashi et al. on 261 babies born between 2005 to 2007 reported no significant differences in the blood IL-6 concentration of infants born by vaginal delivery or cesarean delivery delivery (14).

As a limitation of our study, the influence of antenatal factors was not evaluated and this influence requires testing in a larger population. In conclusion, our study showed that serum IL-6 value in mothers with normal delivery was significantly higher compared to mothers with elective caesarean delivery method (p<0.05).

## Notes

**Funding:** This project was supported by the Vice-Chancellery for Research of Babol University of Medical Sciences.
